# The representation of sound localization cues in the barn owl's inferior colliculus

**DOI:** 10.3389/fncir.2012.00045

**Published:** 2012-07-11

**Authors:** Martin Singheiser, Yoram Gutfreund, Hermann Wagner

**Affiliations:** ^1^Department of Biology, RWTH Aachen UniversityAachen, Germany; ^2^Department of Zoology and Animal Physiology, Institute for Biology II, RWTH Aachen UniversityAachen, Germany; ^3^Department of Physiology and Biophysics, The Ruth and Bruce Rappaport Faculty of Medicine, TechnionHaifa, Israel

**Keywords:** sound localization, central nucleus of the inferior colliculus, auditory, plasticity, adaptation, interaural time difference, interaural level difference, frequency tuning

## Abstract

The barn owl is a well-known model system for studying auditory processing and sound localization. This article reviews the morphological and functional organization, as well as the role of the underlying microcircuits, of the barn owl's inferior colliculus (IC). We focus on the processing of frequency and interaural time (ITD) and level differences (ILD). We first summarize the morphology of the sub-nuclei belonging to the IC and their differentiation by antero- and retrograde labeling and by staining with various antibodies. We then focus on the response properties of neurons in the three major sub-nuclei of IC [core of the central nucleus of the IC (ICCc), lateral shell of the central nucleus of the IC (ICCls), and the external nucleus of the IC (ICX)]. ICCc projects to ICCls, which in turn sends its information to ICX. The responses of neurons in ICCc are sensitive to changes in ITD but not to changes in ILD. The distribution of ITD sensitivity with frequency in ICCc can only partly be explained by optimal coding. We continue with the tuning properties of ICCls neurons, the first station in the midbrain where the ITD and ILD pathways merge after they have split at the level of the cochlear nucleus. The ICCc and ICCls share similar ITD and frequency tuning. By contrast, ICCls shows sigmoidal ILD tuning which is absent in ICCc. Both ICCc and ICCls project to the forebrain, and ICCls also projects to ICX, where space-specific neurons are found. Space-specific neurons exhibit side peak suppression in ITD tuning, bell-shaped ILD tuning, and are broadly tuned to frequency. These neurons respond only to restricted positions of auditory space and form a map of two-dimensional auditory space. Finally, we briefly review major IC features, including multiplication-like computations, correlates of echo suppression, plasticity, and adaptation.

## Introduction

The barn owl (*Tyto alba*) is a nocturnal hunter. These birds are able to approach distant targets under dim illumination and in complete darkness with high accuracy and precision (Payne, 1962, 1971; Konishi, 1973a,b; Hausmann et al., 2008; Singheiser et al., 2010b). They mainly use auditory information for prey capture, and are established as a well-known model system for the study of sound localization. The hearing range of barn owls covers frequencies from 0.2 to 12 kHz. This is rather narrow compared with mammals but broad in comparison with other birds. The lowest thresholds are found between 4 and 8 kHz (Konishi, 1973b; Dyson et al., 1998).

The outstanding auditory capabilities of these animals are mediated by morphological as well as neuronal specializations. For example, the facial ruff functions as a sound collector and amplifier guiding the incoming sound to the ear openings (Payne, 1971; Coles and Guppy, 1988). The facial ruff also enhances the directionality of the ears (Von Campenhausen and Wagner, 2006; Hausmann et al., 2009, 2010). This enhanced directionality improves the representation of the main behavioral cues involved in sound localization: interaural time differences (ITDs) and interaural level differences (ILDs). ITDs change almost exclusively with azimuth. The asymmetrically arranged ears of the barn owl cause ILDs in the high frequency range to change along an axis that is inclined toward the horizontal plane (Moiseff, 1989a,b; Von Campenhausen and Wagner, 2006). The importance of ITDs and ILDs for sound localization was demonstrated in behavioral studies with virtual auditory stimuli replayed to the owls via headphones (Moiseff and Konishi, 1981; Saberi et al., 1998; Egnor, 2001; Poganiatz and Wagner, 2001; Poganiatz et al., 2001; Hausmann et al., 2009, 2010).

The inferior colliculus (IC) is a central processing unit through which almost all auditory information must pass before it can reach the more central nuclei in both mammals and birds (Caird, 1991; Covey and Carr, 2005). The correct anatomical term for the avian homolog of the IC is mesencephalicus lateralis dorsolis (MLd). In line with the owl literature since the study of Knudsen (1983), we shall use the term IC. Much more is known about the mammalian IC than the avian IC. On the other hand the barn owl IC shows some specializations that make it interesting for comparative studies on the anatomy and function of the auditory system. This review concentrates on the morphology and physiology of the barn owl IC. While reviews on specialized issues are available (Wagner, 2004; Takahashi, 2010; Ashida and Carr, 2011), a comprehensive overview over the different aspect of computations in barn owl IC is missing. We shall introduce the auditory pathway and then describe the different sub-nuclei of the IC before we turn to the physiological response characteristics and the relevant computational steps in each sub-nucleus of IC.

## The auditory pathway

The auditory pathway of the barn owl corresponds to the general pathway found in birds. However, the nuclei are enlarged in size (Kubke et al., 2004; Guitiérez-Ibáñez et al., 2011). This holds especially for the IC. The IC is a major relay and processing station for acoustic information and two distinct projections emerge from its central nucleus (Arthur, 2005).

**Figure 1:**
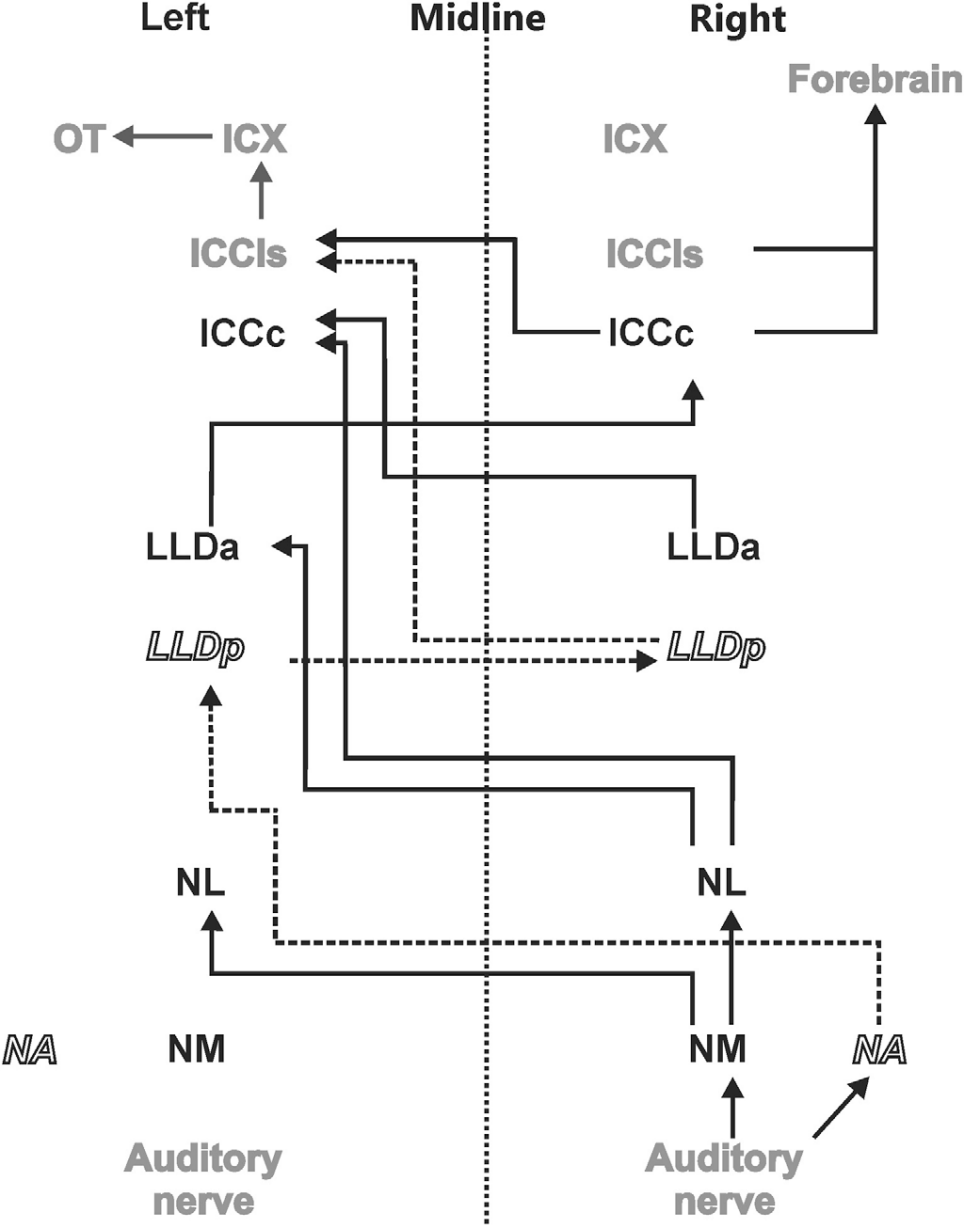
**Auditory pathway.** The afferent auditory pathway is schematically illustrated starting from the auditory nerve upstream to the optic tectum (OT) where the auditory and visual maps merge. Nuclei mentioned in black transmit only ITD information (black solid lines) whereas nuclei indicated in white underlie processing of ILDs and their precursors only (dashed lines and italics). Nuclei shown in gray are involved in the processing of both ITD and ILD information.

The sounds arriving at the two ears are first decomposed into frequency components at the basilar papilla of the cochlea (Köppl et al., 1993). This frequency decomposition creates narrow bandpass filters. The fibers of the 8th nerve receive input from auditory hair cells and send their axons to the cochlear nucleus. In birds, the cochlear nucleus consists of two parts: the laterally positioned nucleus angularis (NA) and the medially lying nucleus magnocellularis (NM) (see Figure 1 for a scheme of the auditory pathway). Each auditory nerve fiber bifurcates into two main collaterals, one of which terminates in NA and the other in NM (Carr and Boudreau, 1991). Both the hair cells and the fibers of the 8th nerve are narrowly tuned to frequency. While the 8th nerve fibers carry information about both the timing and level of the stimulus, information about these two cues splits at the synapses between the 8th nerve fibers and the cells in the cochlear nuclei, so that the neurons of NA carry precise information about stimulus level while cells of NM carry precise information about stimulus timing (Sullivan and Konishi, 1984). In other words, the NA is the starting point of the so called intensity pathway, processing information to compute, and represent ILD, while NM is the first nucleus in the time pathway, processing information to compute and represent ITD (Moiseff and Konishi, 1983). Iso-ITD lines and iso-ILD lines do not run parallel, but are inclined to each other in three-dimensional space. Such a spatial outlay is equivalent to independence in mathematics. The two non-overlapping pathways guarantee independent processing. The information converges in the IC (Adolphs, 1993a), where the construction of a two-dimensional map of auditory space starts (Takahashi et al., 1984).

Information about stimulus level is encoded in the spike rates of NA neurons that exhibit a large dynamic range (Sullivan and Konishi, 1984; Köppl and Yates, 1999). Information from NA reaches the contralateral dorsal lateral lemniscal nucleus pars posterior (LLDp, previously referred to as VLVp, Figure 1). Binaural interactions occur in LLDp and as a consequence, the response of the neurons varies with ILD. Timing information in NM is preserved by locking the temporal occurrence of action potentials to the stimulus phase (Sullivan and Konishi, 1984; Köppl, 1997b). Phase-locking in the barn owl occurs up to frequencies of about 9 kHz (Köppl, 1997a) or at least one octave higher than in other laboratory animals. NM projects to both ipsi- and contralateral nucleus laminaris (NL) (Takahashi and Konishi, 1988a,b). NL is, therefore, the first site in the auditory pathway where input from both hemispheres converges. The axons originating in NM form delay lines and contact arrays of coincidence detector neurons in NL (Carr and Konishi, 1988, 1990). The organization of NL is very similar to what was originally proposed by Jeffress (1948). Coincidence detector neurons fire maximally when information from both sides arrives simultaneously within a short time window. Because the delay lines compensate individual interaural delays in a space-dependent manner, information is converted from a time code into a place code in NL. Laminaris neurons project to both, the contralateral LLDa (previously referred to as VLVa) (Takahashi and Konishi, 1988b) and the core of the central nucleus of the IC (ICCc) (Moiseff and Konishi, 1983). Neurons in the ICCc project contralaterally to the lateral shell of the central nucleus of the IC (ICCls) (Takahashi et al., 1989), where the time and the intensity pathways converge (Adolphs, 1993b). From ICCls, information is sent ipsilaterally to the external nucleus of the IC (ICX), where so called space-specific neurons represent the location of a sound source (Knudsen and Konishi, 1978a) by their combined sensitivities to ITD and ILD. Space-specific neurons in the ICX are arranged in a topographic manner to create a two-dimensional map of auditory space.

From ICX the information is projected to the optic tectum (OT) where the auditory map merges with the visual map from the retina to create a multisensory map of space (Knudsen and Knudsen, 1983). This midbrain pathway (ICC-ICX-OT) is the pathway that subserves precise sound localization (Cohen and Knudsen, 1999; Vonderschen and Wagner, 2009; Singheiser et al., 2010b). An additional pathway, the forebrain pathway, also originates in the ICC. ICC neurons project to the thalamic nucleus ovoidalis (NO) (Proctor and Konishi, 1997; Cohen and Knudsen, 1998; Arthur, 2005). Upstream of NO, information is further propagated to Field L (Cohen et al., 1998) and to the auditory arcopallium (AAr) (Cohen and Knudsen, 1995, 1998). In these nuclei, neurons show a broader frequency tuning than in ICX. Specifically frequencies below 3 kHz, which are missing in the midbrain pathway, are represented (Pérez and Peña, 2006; Pérez et al., 2009; Vonderschen and Wagner, 2009). No map of auditory space could be found in the forebrain (Cohen and Knudsen, 1995, 1996). ITD tuning curves are differently shaped and represent auditory space coarsely (Vonderschen and Wagner, 2009, 2012). Therefore, the forebrain pathway seems to be involved in coarse rather than precise sound localization.

## The morphology of the inferior colliculus

The IC and its subdivisions were originally described by Knudsen (1983). This author distinguished a central nucleus (ICC), an external nucleus (ICX), and a superficial (ICS) nucleus on the basis of their cyto- and myeloarchitecture as well as the connectivity and physiological response properties. By using different stainings (Nissl, Golgi, fiber, and myelin stains), Knudsen (1983) further subdivided the ICC into a dorsal and a ventral part, ICCd, and ICCv, respectively. However, no evidence for functional relevance of the latter differentiation has been found so far. Knudsen (1983) also noted that auditory information ascending from the lateral lemniscal nuclei exclusively enters the ICC, and not ICX or ICS.

In a different approach Takahashi and Konishi (1988a) used anterograde (tritiated [^3^H]-proline) and retrograde [horseradish peroxidase (HRP)] labeling to uncover the projections of lower brainstem nuclei like NM, NL, and NA to the IC. Labeling of the rostral part of ICC was observed after injections of [^3^H]-proline into NL. The horizontal level of labeled fibers depended on the injection site along the tonotopic axis of NL: when proline was injected in low best frequency regions of NL, the staining occurred in dorsal regions of ICC whereas proline injections in high frequency regions resulted in more ventral staining in ICC giving a hint of a tonotopy of frequencies that is maintained from NL to ICC (see Takahashi and Konishi, 1988a; their Figure 8). In contrast to the tonotopy, no evidence for topography could be found in ICC, when proline was injected at different dorso-ventral positions at similar antero-posterior positions of NL: the labeling in ICC was indistinguishable from each other. In retrospect it seems that these injections might have been too large, because it is now known that NL contains a map of ITD in its dorso-ventral extent (Sullivan and Konishi, 1986) that is reflected in an equivalent map in the antero-posterior direction in ICC (Wagner et al., 1987).

When Takahashi and Konishi (1988a) analyzed the labeling in ICC produced by injections of proline, they observed that the terminal field of NL in ICC forms a vertical “core” restricted to the rostral 40% of the ICC. Their core-region involved both the laminated as well as the non-laminated parts of ICC that were previously described by Knudsen (1983). Injecting the retrogradely transported HRP into the ICC resulted in labeled somata predominantly in NL. The sub-nucleus of the ICC that receives NL input was termed the ICCc.

When proline was injected into the NA of the owls, it was anterogradely transported to the contralateral ICC as well as to other brainstem nuclei like the contralateral LLDp (VLVp). [^3^H]-proline labeling in ICC was present throughout the nucleus. While caudally the whole nucleus was labeled, from about 0.4 of the total length of IC in reference to the caudal pole, the labeled field split in two regions. Takahashi and Konishi termed this region the “shell” of the ICC (for further details see Takahashi and Konishi, 1988a, their Figure 13). Nowadays, the shell of the ICC is further divided into a medial shell (ICCms) and a lateral shell (ICCls) (Takahashi et al., 1989; Wagner et al., 2003). Takahashi and Konishi (1988a) demonstrated that the terminal fields of NA and NL seem to be largely non-overlapping, supporting the hypothesis that ITD (via NL to ICCc) and ILD (via LLDp to ICCls) were processed in parallel non-overlapping pathways.

**Figure 2:**
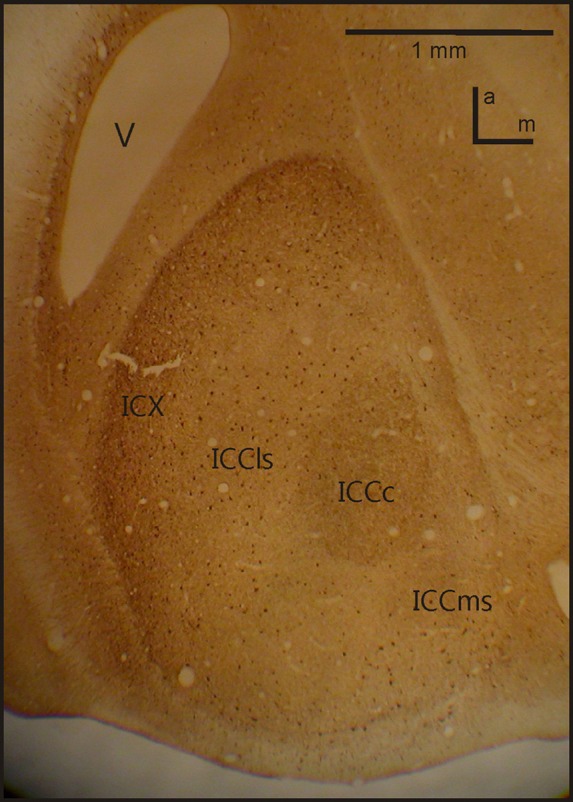
**IC anatomy.** A horizontal section through IC with the four important sub-nuclei. Photograph from a staining with Calbindin. *V*, ventricle; *a*, anterior; *m*, medial.

A further study on the anatomy of the IC subdivisions and neighboring structures was carried out by Wagner et al. (2003). These authors used antibodies directed against several substances (e.g., tyrosine hydroxylase, γ-amino butyric acid (GABA)_A_β, dopamine- and cyclic AMP regulated phosphor protein (DARPP-32), calretinin and calbindin) as well as somata staining with cresyl violet and fiber staining using the Gallyas procedure. Wagner et al. (2003) discriminated eight different structures belonging to the three IC subdivisions (ICC, ICX and ICS) as well as the neighboring OT. The periventricular tectal layers 15a and 15b could be well stained with all antibodies used in the study. All antibodies tested in the study sufficiently marked the boundary between the tectal layer 15a and the ICX. The DARPP-32 antibody caused much more darkly labeled somata in ICX than in ICCls. In addition Rodriguez-Contreras et al. (2005) demonstrated exclusive expression of calcium/calmodulin-dependent protein kinase II in ICX. Antibodies against calbindin and calretinin clearly marked the neurons of ICCc (Figure 2). None of the antibodies was sufficient to delineate the border between ICCls and ICCms.

## The core of the central nucleus of the inferior colliculus (ICCc)

### Tuning properties of ICCc neurons

**Figure 3:**
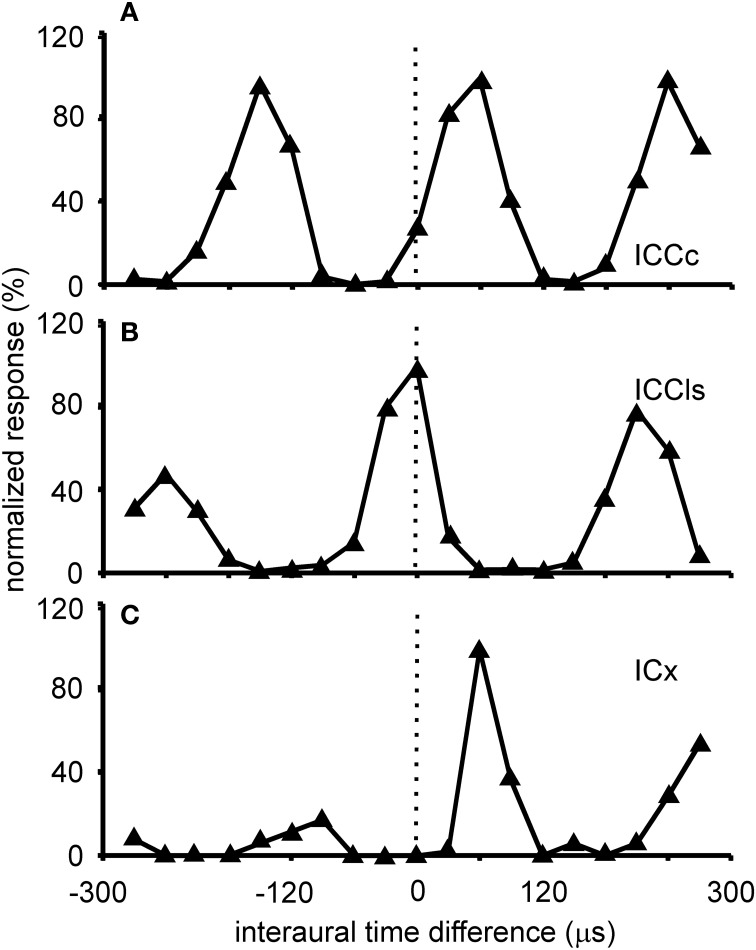
**ITD tuning.** Shown are examples of the variations of the normalized responses with ITD of neurons located in different sub-nuclei of the IC. The stimulus for collecting these tuning curves was broadband noise. ITD tuning is periodic in the ICCc with the distance of the peaks reflecting the best frequency of the neuron **(A)**. All peaks have a similar height. A similar tuning is observed in the cells of the ICCls **(B)**. By contrast in the responses of the cells of the ICX one peak, the main peak, is clearly dominant compared to the other peaks, the side peaks **(C)**.

ITD tuning is observed across the entire dorso-ventral extent of ICCc (Wagner et al., 1987, 2002, 2007; Takahashi et al., 1989; Wagner and von Campenhausen, 2002; Bremen et al., 2007). The responses of the neurons in ICCc are phase ambiguous within the physiological range of ITDs (Figure 3A). This response pattern occurs to stimulation with both broadband noise as well as with pure tones. The heights of the peaks in the ITD tuning curve are all comparable. Thus, it is impossible to discriminate a main peak from the side peaks when information about only a single neuron is available (for a possible discrimination in the case of many neurons tuned to different frequencies, see below). Since all peaks have similar heights, side peak suppression does not occur in these neurons. The distance between the ITD peaks corresponds to about 1/best or characteristic frequency (BF/CF) for noise/pure tone stimulation (Wagner et al., 1987, 2002, 2007; Wagner, 1990; Fujita and Konishi, 1991). Due to this cyclic tuning with similar heights of the ITD peaks, ICCc neurons represent the phase difference between sounds arriving at the left and right ears. Since these phase differences correspond to more than one ITD, ICCc neurons show phase-ambiguity to ITD stimulation and cannot signal the azimuthal position of the sound source unambiguously. Note that this holds, although the mechanism underlying the detection of the phase difference is based on delays (Wagner, 2004; Ashida and Carr, 2011). The core of the ICC is tonotopically organized, with low frequencies represented in the dorsal region of the nucleus and high frequencies ventrally (Wagner et al., 1987, 2002, 2007; Takahashi et al., 1989). When electrode penetrations are made perpendicular to iso-frequency laminae in ICCc, recorded neurons share one common best ITD, the so called array-specific ITD. The array-specific ITD corresponds to the slope of the regression line in phase-frequency plots and represents ipsilateral locations in ICCc (Wagner et al., 1987, 2007). The array-specific ITD also corresponds to the main peak in the ITD tuning curve in most cases. Furthermore, array-specific ITDs are mapped and represent the entire auditory space of the barn owl (Wagner et al., 1987, 2007; Takahashi et al., 1989).

**Figure 4:**
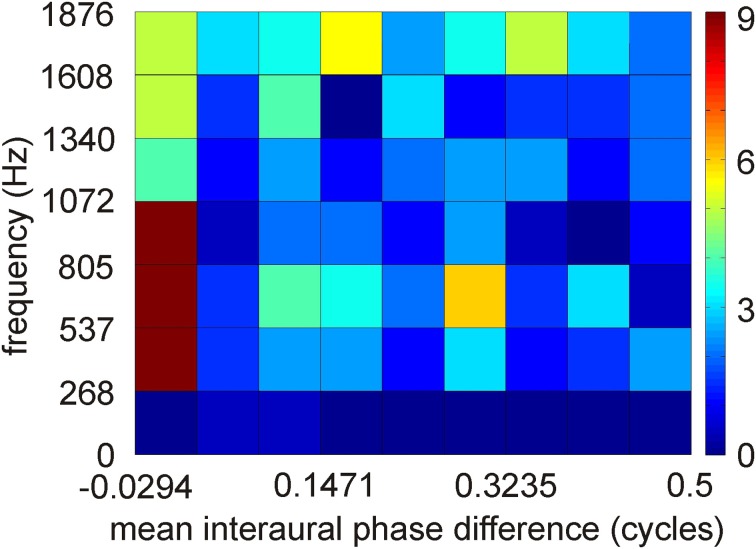
**Distribution of interaural phase difference in low-best frequency neurons of ICCc.** Distribution of 251 data points. Interaural phase differences were calculated from interaural time differences by first restricting the mean interaural phase differences to a range from −0.5 to 0.5 cycles by phase wrapping and then take the absolute value. A binwidth of 0.0625 and of 268 Hz was chosen to make the data comparable to the model data of Harper and McAlpine (2004).

ITD coding in the IC of mammals differs from that observed in the IC of the barn owl (McAlpine et al., 2001; Wagner et al., 2002, 2007; Harper and McAlpine, 2004). In small mammals, the steepest slopes of many ITD curves cross 0 μs ITD, while this is not the case in the barn owl. Harper and McAlpine (2004) proposed a model of optimal ITD representation. According to this model, the representation of ITD depends on frequency. The representation above a frequency whose period is smaller than twice the physiological ITD range (about 2–2.5 kHz in the barn owl) is map-like. The experimental data collected from the high frequency region in the barn owl's IC are in agreement with this theory. By contrast, certain interaural phase differences are preferred for lower frequencies in the model. For example, the model did not produce any data points from −0.5 to −0.25 and +0.25 to 0.5 mean interaural phase difference in the low-frequency range. The data from ICCc in the low-frequency region show a distribution with a preference for 0 mean interaural phase difference (Figure 4). The similarity with the theoretical prediction is limited, if the plot shown in Figure 4 is compared with the corresponding plot (Figure 2D) in the paper by Harper and McAlpine (2004). A two-dimensional cross-correlation between the model data and the experimental data revealed that not more than 20% of the data can be explained by the theory. This low explanatory power of the model seems to be mainly due to the existence of experimental data points from −0.5 to −0.25 and from 0.25 to 0.5 mean interaural phase difference which are absent in the model data. Additionally, best ITDs were observed that not only lie outside the physiological ITD range (Wagner et al., 2002, 2007; Singheiser et al., 2010a) but also outside the so called π-limit (Wagner et al., 2007). The functional significance of the ITDs outside the physiological ITD range in the barn owl remains elusive. Neither the model of slope coding (McAlpine et al., 2001; Brand et al., 2002; Hancock and Delgutte, 2004; Harper and McAlpine, 2004; Siveke et al., 2006; Wagner et al., 2007) nor the stereausis model (Singheiser et al., 2010a) could account for these peaks.

**Figure 5:**
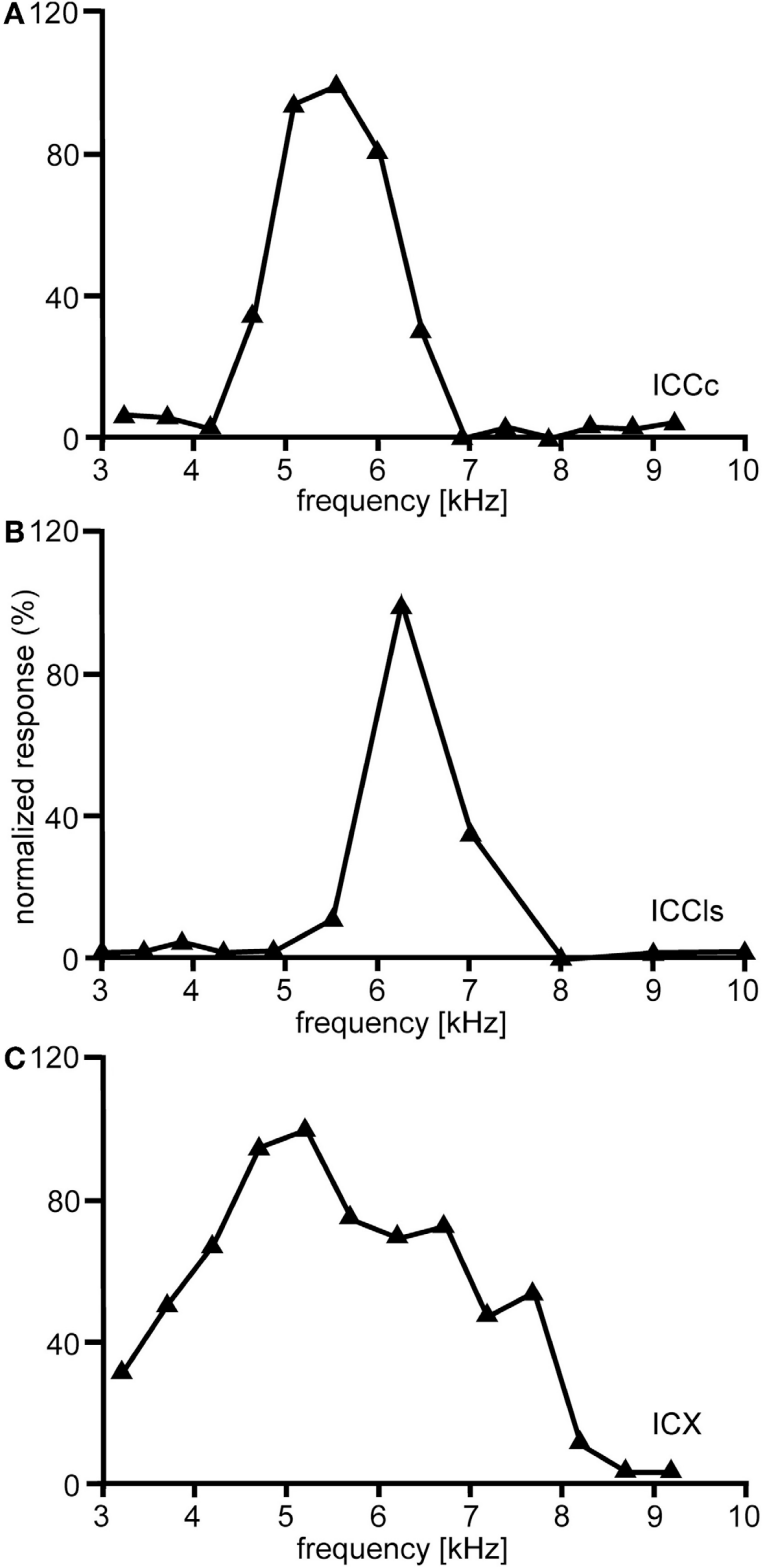
**Frequency tuning.** Shown are examples of the variations of the normalized responses with frequency of neurons located in different nuclei of the IC. In ICCc frequency tuning is narrow **(A)**. A similar tuning is observed in the cells of ICCls **(B)**. By contrast frequency tuning of the cells of the ICX is broader than in ICC **(C)**.

The entire frequency spectrum of the barn owl's hearing range (0.2–12 kHz, Wagner et al., 2002, 2007) is represented in ICCc. Iso-intensity frequency tuning curves (hereafter referred as to frequency tuning curves) are typically symmetric and narrowly tuned (Q_10 dB_ = 1–14; Knudsen, 1984), single peaked with steep slopes on both the low- and high frequency flanks of the peak (Figure 5A). Frequency tuning widths measured at half-height of the frequency tuning curves are positively correlated with BF for both binaural (Wagner et al., 2002) and monaural stimulation. Nonetheless, the quality factor of frequency tunings (ratio of tuning width to BF) decreases with BF (Wagner et al., 2002). Generally, frequency tuning widths are on average one octave for neurons having BFs <1 kHz and about 1/3 octave for neurons having higher frequencies (Wagner et al., 2002). Moreover, no significant differences between BFs in the ipsi- and contralateral inputs of ICCc have been observed, at least for neurons in the low-frequency range (Singheiser et al., 2010a).

**Figure 6:**
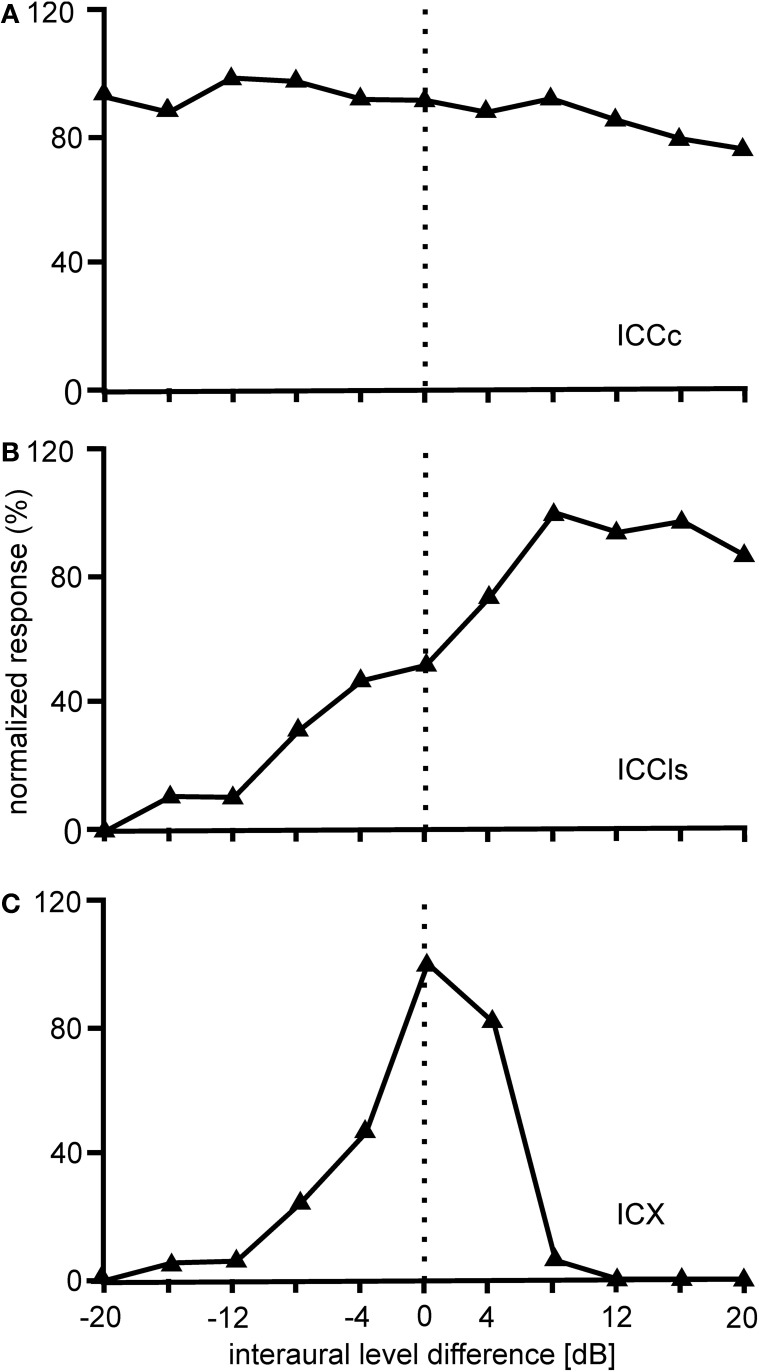
**ILD tuning.** Shown are examples of the variations of the normalized responses with ILD of neurons located in different nuclei of the IC. The responses of ICCc cells do not vary with the interaural level difference (ILD) **(A)**. The ILD tuning of the cells in ICCls is sigmoidal **(B)**, while the ILD tuning in ICX is bell-shaped **(C)**.

Further tuning characteristics of ICCc neurons will be mentioned briefly in the following. ILD tuning curves in ICCc are flat (Figure 6A), while rate-vs.-level functions (RLF) in ICCc are primary-like (Wagner et al., 2002; Bremen et al., 2007). Input to both the ipsi- and the contralateral ear excites ICCc neurons (EE property). Responses to ipsi- and contralateral stimulation do not differ in threshold, slope, dynamic range, or saturation level (Wagner et al., 2002).

### Connectivity of ICCc and further physiological properties

The ICCc receives its inputs directly from the contralateral NL (Takahashi and Konishi, 1988a) and indirectly from the ipsilateral NL via the contralateral LLDa (Adolphs, 1993a). Whereas NL neurons require averaging of responses derived from multiple presentations for each ITD to arrive at a veridical representation (Christianson and Peña, 2006; Fischer and Konishi, 2008), in the ICCc a single presentation of ITD is sufficient for a reliable ITD response, showing a profound noise reduction in the ascending auditory pathway (Christianson and Peña, 2006). Phase-locking is also dramatically reduced in the projection from NL to ICCc. This loss of fine temporal information in the ongoing spike train is replaced by envelope coding of the signal, preserving the temporal information of the ITD pathway in the temporally integrated responses (Christianson and Peña, 2007).

The ICCc has two ascending projections: one reaches the contralateral ICCls, endowing it with ITDs from the contralateral hemifield, as first shown by both retrograde (HRP) and anterograde (tritiated proline) labeling of neurons in ICCls and ICCc, respectively (Takahashi et al., 1989). As mentioned above, this projection is the beginning of the midbrain pathway that continues via ICX to the OT. The second projection goes upstream via the thalamic NO to higher order nuclei (Cohen et al., 1998; Arthur, 2005) and is the beginning of the forebrain pathway.

## The lateral shell of the central nucleus of the inferior colliculus (ICCLs)

### Tuning properties of ICCLs neurons

Responses of ICCls neurons to varying ITDs are generally comparable to those recorded in ICCc: when stimulated either with pure tones or broadband noise, neurons in ICCls show a cyclic tuning, where the side peaks are almost as high as the main peak. Sidepeak suppression with broadband stimulation is absent or very weak (Gold and Knudsen, 2000) (Figure 3B). Thus, the responses are phase ambiguous. This phase-ambiguity occurs irrespective of stimulus bandwidth, due to the narrow frequency tuning curves in ICCls. Furthermore, the distance between the peaks is again characterized by integer multiples of the best frequency (Wagner et al., 1987, 2007; Wagner, 1990; Fujita and Konishi, 1991; Bremen et al., 2007). In contrast to ICCc, however, where the array-specific ITD represents ipsilateral locations, array-specific ITDs in ICCls represent locations in contralateral space (Wagner et al., 1987, 2007; Takahashi et al., 1989).

The frequency tuning of the neurons in ICCls is similar to that seen in ICCc (compare Figure 5A with Figure 5B). Frequency-tuning width is narrow to intermediate (Takahashi and Konishi, 1986; Wagner et al., 1987, 2007). The width of frequency tuning curves varies both with best frequency (Wagner et al., 2002) and with the location within ICCls (Mazer, 1998). Neurons located more laterally have wider tuning than neurons located more medially. A clear tonotopy covering the entire frequency range of the barn owl is found in the dorso-ventral extension of ICCls. However, a smaller proportion of neurons in ICCls than in ICCc have best frequencies below 2.5 kHz (31.1% in ICCc and 17.4% in ICCls) and frequencies below 0.8 kHz were extremely rare (Wagner et al., 2007). As a consequence of missing very low best frequencies, the ITD range in ICCls is narrower than that in ICCc (750 μs vs. 1500 μs, respectively; Wagner et al., 2007).

The ICCls is the first station in IC where ILD information is present. ILDs from the contralateral LLDp are added (Takahashi and Konishi, 1988b; Takahashi and Keller, 1992; Adolphs, 1993b) to the ITD information ascending from the time pathway (Figure 6B). Data from Adolphs (1993a) suggested the presence of a bilateral ILD projection from LLDp to ICCls. Using pharmacological manipulations to increase or decrease the neural activity of LLDp, Adolphs (1993a) demonstrated that LLDp provides direct functional GABAergic inhibition to ICCls neurons. Tuning curves to varying ILDs in ICCls show a sigmoidal or open peaked characteristic favoring contralateral-ear louder ILDs (Adolphs, 1993a; Bremen et al., 2007). Monaural responses show an EI pattern: excitation to contralateral stimulation and inhibition to ipsilateral stimulation (Adolphs, 1993a; Wagner et al., 2002).

Auditory responses in the ICCls are mediated by a family of excitatory amino acid receptors of which the most prominent are NMDA and non-NMDA glutamate receptors. By applying the NMDA receptor antagonist 2-amino-5-phosphonovaleric acid (AP5) as well as the non-NMDA receptor antagonist 6-cyano-5-nitroquinoxaline-2,3-dione (CNQX), responses in ICCls could be altered: while AP5 reduced the stimulus-evoked responses significantly in only less than 50% of the recording sites, CNQX application strongly reduced responses in most ICCls sites (Feldman and Knudsen, 1994). Thus, non-NMDA currents seem to mediate most auditory responses in ICCls.

### Output projections of ICCLs

The ICCls has two output targets (Figure 1). One projection parallels the thalamic output of ICCc neurons to NO and higher brain centers (Cohen et al., 1998; Arthur, 2005). The second projection reaches the ipsilateral ICX and serves to eliminate phase ambiguities and to compute the spatially restricted receptive fields of auditory neurons in ICX (Wagner et al., 1987).

## The external nucleus of the inferior colliculus (ICX)

The responses of ICX neurons to pure-tone ITD stimulation are similar to those of the neurons in ICC, in that all response peaks have a similar height (Takahashi and Konishi, 1986; Fujita and Konishi, 1991; Wagner, 1993). However, when broadband noise is used as stimulus, the ITD tuning curve, although still cyclic, is characterized by a dominant main peak and smaller side peaks (Takahashi et al., 1984; Takahashi and Konishi, 1986; Wagner, 1990, 1993; Fujita and Konishi, 1991) (Figure 3C). In other words, strong sidepeak suppression is typically observed in these neurons when they are stimulated with broadband noise. In the ICX, ILD-tuning curves are generally bell shaped (Takahashi et al., 1984) (Figure 6C), and the values of the best ILDs span the range from about −15 to +15 dB. Frequency tuning is broader in ICX than in ICC (compare Figure 5C with Figures 5A,B) with Q_10 dB_ values of frequency tuning in ICX lying between 1 and 4 (Knudsen, 1984). Unlike in ICC, in the ICX there is almost no representation of best frequencies below 2.5 kHz (Knudsen and Konishi, 1978a; Mazer, 1998; Wagner et al., 2007). Monaural stimulation or binaurally uncorrelated stimulation elicits only weak responses in ICX neurons (Takahashi et al., 1984, 1989; Albeck and Konishi, 1995).

### The computation of auditory space—elimination of phase ambiguities

To unambiguously represent the position of a sound source in the horizontal plane, phase ambiguities have to be eliminated. Both electrophysiological recordings in the barn owl's ICX (Mazer, 1998) and OT (Saberi et al., 1998) and behavioral experiments using head turning movements (Saberi et al., 1999) have demonstrated that the a signal bandwidth of at least 3 kHz is needed for an unambiguous signaling of the position of a sound source in azimuth. To do so, convergence of neurons tuned to different BFs onto a single neuron or a small cluster of neurons is required. This converging projection is realized at the synapses between ICCls and ICX: narrowly tuned ICCls neurons that respond to different BFs in a column of an array-specific ITD project onto single neurons located in ICX which consequently become broadly tuned to frequencies but signal the same best ITD (Takahashi and Konishi, 1986; Wagner et al., 1987). This frequency convergence enhances the response to the “true” position of the sound source compared with the responses to phase equivalent positions in a bandwidth-dependent manner; the broader the tuning, the higher sidepeak suppression (Mazer, 1998). However, this frequency convergence is not the only mechanism underlying sidepeak suppression. Peña and Konishi (2000) recorded intracellularly from space-specific neurons in ICX and found that the difference between the main and the side peaks is smaller in postsynaptic potentials than in spike counts. This finding led to the conclusion that ICX neurons themselves enhance side peak suppression when converting membrane potentials into spikes. A third factor in side peak suppression is GABAergic inhibition that acts on the side peaks and thus enhances the main side peak ratio (Fujita and Konishi, 1991; Albeck, 1997; Mori, 1997; Peña and Konishi, 2000, 2002). When GABAergic inhibition was blocked by the antagonist bicuculine methiodide, ICX neurons no longer signaled only the main ITD but also responded to its phase-equivalents (Fujita and Konishi, 1991).

### Space-specific neurons and the map of auditory space

**Figure 7:**
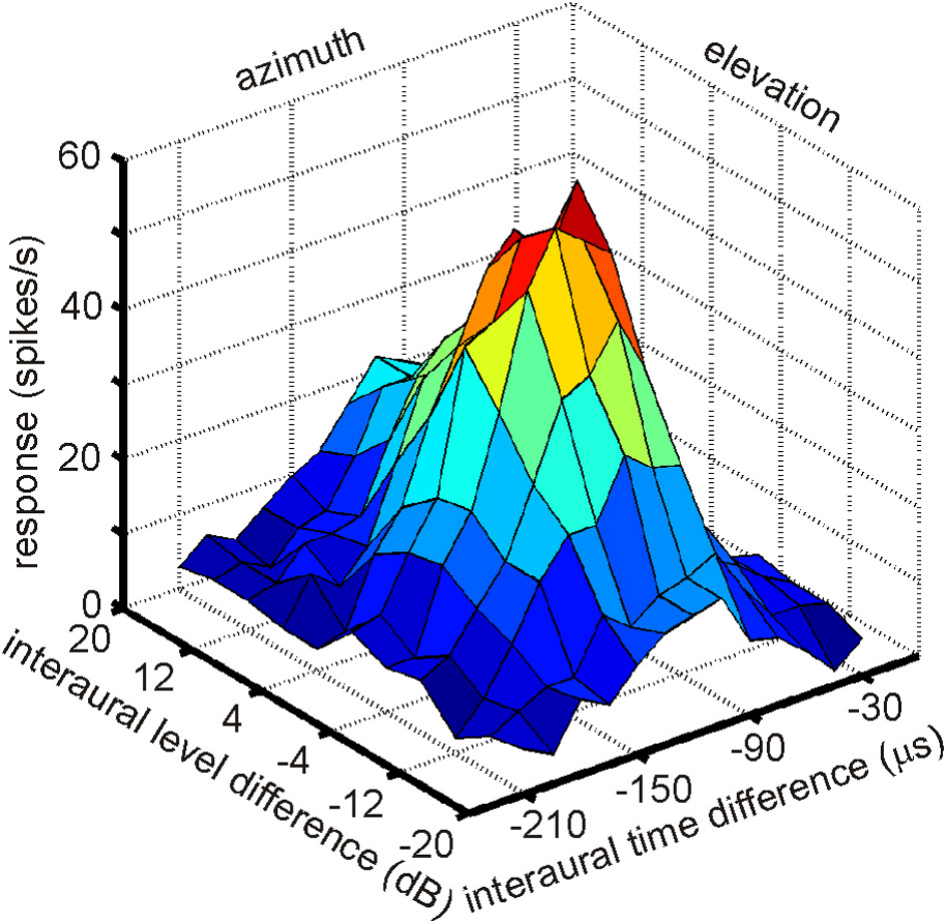
**A multiplicative receptive field of an ICX neuron.** The response of this cell depends on ITD, which correlates with azimuth and on ILD, which correlates with elevation. Note that the responses in the two orthogonal directions, azimuth and elevation, are independent, because the shape of the curve of one parameter remains constant as the other parameter is changed. Only the height of the peak changes.

The existence of a physiological map of auditory space in IC was first demonstrated by Knudsen and Konishi (1978a). Using a combination of free field stimulation with moveable speakers and extracellular recordings in the barn owl's IC, these authors were able to demonstrate that neurons in the lateral region of the IC, today known as the ICX, responded only when the sound was within a well circumscribed region of auditory space. This spatial receptive field was later found to be defined by a best ITD and best ILD determining the horizontal and elevational position of the receptive field, respectively. The level of the stimulus had little effect on the location of a receptive field. These neurons were termed space-specific neurons. When Knudsen and Konishi (1978a) plotted the receptive fields of space-specific neurons on a globe it turned out that the receptive fields of neighboring units overlapped. Furthermore, when plotting the receptive fields of neurons sequentially recorded along a dorso-ventral penetration of the electrode the fields shifted in elevation from high to low. Elevational positions covered the vertical space spanning from +20° to −90°. Moreover, the receptive fields of space-specific neurons in the ICX shifted from frontal space in the rostral parts of ICX to far contralateral space in the caudal portions of the ICX. Thus, azimuthal space was mapped along the rostro-caudal axis of ICX while elevational space was mapped along the dorso-ventral axis. The frontal azimuthal space between ±20° was overrepresented (Knudsen and Konishi, 1978a,b). While the initial study was based on only one animal and only 19 neurons, many subsequent studies have confirmed the original finding (Wagner et al., 1987; Brainard and Knudsen, 1993; Hyde and Knudsen, 2000; DeBello et al., 2001; Bergan et al., 2005). In a further experimental study on the tuning characteristics in ICX, Takahashi et al. (1984) demonstrated the independence of timing and intensity cues to create space-specific neurons. When NA was silenced with lidocaine, ILD tuning curves of space-specific neurons in ICX were shifted, whereas ITD tuning curves remained stable. When NM was temporally knocked out, ITD tuning curves changed while ILD tuning curves remained unaltered. Furthermore, the best ITD was independent of the ILD of a stimulus (Takahashi et al., 1984; see Figure 7 for an example). The same effect was found for ILD tuning curves recorded with different ITDs. In a third study on the organization of space-specific neurons and the encoding of auditory space in the barn owl's ICX, Knudsen and Konishi (1978c), again using free-field stimulation and extracellular recordings, reported “two functionally antagonistic areas”: whereas the center of the receptive field was excitatory, the surround was inhibitory. The inhibitory surround of the receptive field was strongly dependent on the location of inhibition. The inhibitory surround was stronger for the azimuthal than the elevational direction which well matched the higher resolution of space-specific neurons in the horizontal plane.

The relevance of space-specific neurons for precise sound localization was demonstrated by small lesions in the auditory space map (Wagner, 1993). Head movements of three owls were recorded before and after lesioning. Whereas the head turns were precise before the lesions were made, head turns were not related to stimulus position after the lesions. The failures in precise head turns could be predicted by the azimuthal position of the lesion site in the space map.

More than 20 years after the first experiments by Knudsen and Konishi (1978a); Peña and Konishi (2001, 2002) as well as Fischer and colleagues (Fischer et al., 2007, 2009) investigated the computations underlying the creation of spatial receptive fields with intracellular recordings and mathematical models. Peña and Konishi (2001) asked how post-synaptic potentials of neurons carrying both ITD and ILD information interact to produce sub- and suprathreshold responses to certain combinations of ITD and ILD. They observed that some ITD-ILD combinations elicited suprathreshold depolarizing postsynaptic potentials whereas other combinations caused hyperpolarization. Such observations are in accordance with the reports of Knudsen and Konishi (1978c) of an excitatory center and an inhibitory surround of space-specific neurons. By careful analysis of the subthreshold responses, Peña and Konishi (2001) confirmed that the inputs of ITD and ILD onto an ICX neuron are independent of each other, consistent with the data of Takahashi et al. (1984). This independence is also obvious from the response profile shown in Figure 7 in which the shape of ILD tuning is independent of the ITD and vice versa. Peña and Konishi (2001) also demonstrated that a multiplicative model for ITD/ILD integration fitted the postsynaptic potential data better than an additive model. Spiking thresholds changed gradually with ongoing stimulus presentation so that the thresholds for the first spikes were lower than those for later spikes. As a consequence, thresholding can sharpen the tuning over time, as had also been suggested by Wagner (1990). While these studies demonstrated that multiplicative processes take place in the integration of ITD and ILD information, they did not resolve where in the auditory pathway this is happens. Fischer and coworkers (2007) recorded extracellularly in ICCls. The majority of the responses (61%) were well described by the multiplicative model, suggesting that ICCls is the first nucleus where multiplicative responses occur. Under natural conditions, ILDs change in a frequency-specific manner, especially for high frequencies, while ITDs are largely frequency-independent (Coles and Guppy, 1988; Keller et al., 1998; Hausmann et al., 2010). Fischer et al. (2009) developed a model, which included natural listening conditions. These authors proposed that multiplication between ITD- and ILD-dependent signals only occurs within frequency channels, and that frequency integration is based on a linear-threshold mechanism. This mechanism allows the system to represent multiple sound sources with natural sound localization cues. The authors concluded that non-linear responses in the owl's IC can be generated by a combination of cellular and network mechanisms.

Bala et al. (2003) investigated the relationship between the barn owl's sound-localization acuity and the neuronal activity rates of space-specific neurons. The pupillary dilation response served as a criterion for behavioral acuity. The pupillary dilation response is a common response in all vertebrates elicited by events that deviate from the common input stream. The response to an auditory stimulus from a given location was habituated, before the stimulus changed location, resulting in an increase of the pupillary dilation response. The threshold value of the spatial change of a sound source that caused the pupillary dilation response to change was about 3°. The change of activity of the space-specific neurons reflected this threshold, leading to the conclusion that behavioral discrimination performance of the barn owl is realized by a change in the activity of a population of space-specific neurons in ICX.

### Sound localization in echoic environments—a possible role of space-specific neurons

Under natural conditions, sounds directly arriving from a single source are generally followed by successive echoes from different locations in space. The system's ability to localize the leading but not the lagging sound is due to the so called precedence effect, meaning the leading sound dominates later arriving reflections of the sounds (for a review see Blauert, 1997). The Takahashi lab (Takahashi and Keller, 1994; Keller and Takahashi, 1996a,b, 2005; Spitzer et al., 2003, 2004; Spitzer and Takahashi, 2006; Nelson and Takahashi, 2008, 2010) has investigated the precedence effect and the underlying mechanisms for precise sound localization in simulated echoic environments in the barn owl by using both behavioral paradigms and electrophysiological recordings. When identical sounds are presented simultaneously from two different positions in space, owls perceive a single sound source in the middle. This behavior is due to “summing localization” (Takahashi and Keller, 1994; Keller and Takahashi, 1996a). Under natural conditions the direct sound leads the echo by a few milliseconds. The leading sound dominates the perception responsible for precise sound localization; this effect is also known as localization dominance. As the delay between lead and lag increases, the lag can be more easily localized. The echo-threshold is defined as the minimal delay required for the perception of a lagging sound. Several studies in barn owls have shown that they turn their head toward leading sound source if the interval between lead and lag is shorter than 10 ms. When the delay between lead and lag increases, the number of head saccades to the lag increase (Keller and Takahashi, 1996b; Spitzer and Takahashi, 2006; Nelson and Takahashi, 2008), accompanied by a reduced ability to signal changes in the locations of simulated echoes (Spitzer et al., 2003). The neural basis for this behavior was found in the responses of the space-specific neurons of the ICX (Keller and Takahashi, 1996b; Spitzer et al., 2004; Nelson and Takahashi, 2008). Not only does the temporal segregation between two stimuli influence localization dominance, but also differences in frequency spectrum, amplitude modulations, and ILDs between the sound and its echoes play a role (Takahashi and Keller, 1994; Keller and Takahashi, 1996a, 2005; Nelson and Takahashi, 2008, 2010).

## Plasticity in the barn owl's inferior colliculus

Several studies have demonstrated that early auditory experience during sensitive periods of maturation has profound effects on the tuning properties of neurons to binaural stimuli like ITD and ILD. Manipulation of spatial cues was achieved by plugging one ear (Mogdans and Knudsen, 1992, 1993, 1994a,b), by implanting a passive filtering device in the ear canal (Gold and Knudsen, 1999, 2000; Miller and Knudsen, 2001, 2003) or by raising barn owls in broadband masking noise (Efrati and Gutfreund, 2011). After about two months of manipulation, the responses to ITD and ILD were recorded in different nuclei of the auditory pathway (ICC, ICX, OT, and NO) and compared to units recorded in control animals. All of the above manipulations induced large changes in the tuning to binaural cues in higher order nuclei [OT (Mogdans and Knudsen, 1992; Gold and Knudsen, 1999; Efrati and Gutfreund, 2011), ICX (Gold and Knudsen, 2000, 2001), NO (Miller and Knudsen, 2003)]. Noise rearing resulted in broader ILD and ITD tuning curves and atypical asymmetrical ILD tuning curves in the OT. Monaural ear-devices or earplugs resulted in frequency-specific changes in the patterns of binaural cues. Changes were in all cases in the adaptive direction, i.e., in the direction that compensates for the distorted spatial cues. For example, monaural occlusion led to shifts of the receptive field of space-specific neurons depending on the side of the earplug: when the left ear was plugged, receptive fields were shifted left- and downward (Knudsen and Konishi, 1980). Thus, space-specific neurons are shaped by experience allowing frequency specific compensation for individual differences as well as unexpected changes in the mapping between auditory cues and space.

In contrast to the large-scale changes of tuning curves found in the ICX, NO, and OT, ICC tuning curves were rather insensitive to the manipulations mentioned above. This suggested that the connections between the ICCls and the ICX are the site of experience-dependent plasticity. The ICCls-ICX projection is the pathway where information across frequency channels converges to create space-specific neurons. This connection is highly topographic in normal owls resulting in a mapped organization of ITDs in the ICX, which is further topographically relayed to the OT (Feldman and Knudsen, 1997; Knudsen and Knudsen, 1983).

The importance of the ICCls-ICX projections in experience-dependent plasticity has been best demonstrated by raising young barn owls with displacing prisms, which shift the visual field horizontally either to the left or to the right (Knudsen, 2002). Barn owls cannot move their eyes by more than a few degrees from their resting positions. Therefore, prisms produce a chronic discrepancy between visual and auditory spatial information. The immediate effect of prisms is an optical displacement of visual space but no effect on auditory space. As a result a mismatch occurs between the internal representation of auditory and visual space. Experience with the prism spectacles over a period of about two months, leads to a horizontal shift in auditory spatial tuning in the OT that realigns auditory receptive fields with the optically displaced visual receptive fields (Knudsen and Brainard, 1991). This is achieved by a systematic shift of ITD tuning curves.

**Figure 8:**
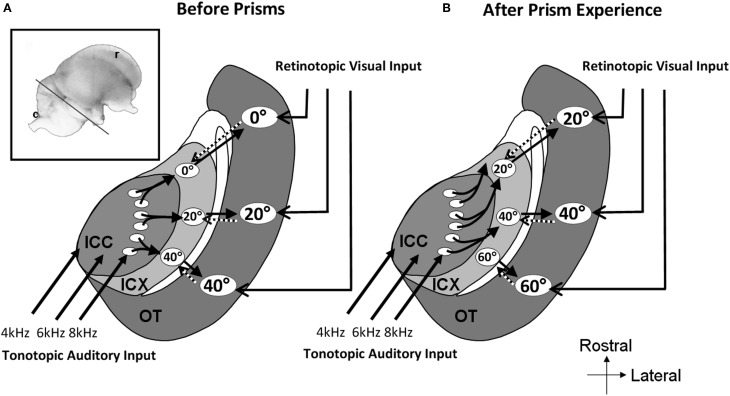
**Schematic representation of the mid-brain auditory localization pathway in normal and in prism-reared owls.** The inset shows a lateral view of the barn owl's brain. The line marks the approximate plane of the section through the tectal lobe illustrated schematically in **A** and **B**. **(A)** Information flows from the central nucleus of the inferior colliculus (ICC) to the external nucleus of the inferior colliculus (ICX) and from there to the optic tectum (OT). A map of auditory space is created in ICX. This map joins the visual retinotopic map arriving from the retina and forebrain in OT. Topographic connections from the OT to the ICX presumably carry spatial visual information to instruct auditory plasticity in the ICX (dotted arrows). The circled numbers in the ICX and OT represent the azimuthal positions in space to which the neurons are tuned to. **(B)** Following a period of several weeks of prism adaptation the axonal connections between the ICC and the ICX grow in an abnormal pattern. Connections are shifted to the rostral direction in one side of the brain and to the caudal direction in the other side of the brain. This pattern of axonal re-growth shifts the auditory maps in the ICX and the OT to align with the shifted visual map.

In prism-reared owls, the auditory space map in the ICX was shifted as well as the tectal map (Brainard and Knudsen, 1993). In contrast, the representation of ITD in the ICCls remained normal (Brainard and Knudsen, 1993). This again pointed to the connection between the ICCls and the ICX as the site of plasticity. Indeed, changes in the anatomy of the axonal projections from the ICCls to the ICX have been observed (Feldman and Knudsen, 1997; DeBello et al., 2001). In prism reared owls the projection is asymmetrically broader than normal. Thus, as a result of prism experience, axons projecting from the ICCls are systematically shifted in the direction that supports the shift of the ITD tuning curves in the ICX (Figure 8).

Pharmacological experiments in prism-reared owls also demonstrated that the ICX is a site of plasticity. Excitatory transmission in the ICX is glutamatergic and relies heavily on NMDA receptor currents (Feldman and Knudsen, 1994). Blocking NMDA-receptors in the ICX selectively by focal application of the NMDA receptor blocker AP5 caused a reduction of about 50% of the normal auditory response. However, in owls that have been exposed to prisms, newly learned responses were far more sensitive to NMDA receptor blockade (Feldman et al., 1996). These data indicated that newly functional synapses in the ICX, supporting shifted responses, are richer in NMDA-receptors. On the other hand, in prism reared owls, responses to normal ITDs were far more suppressed by GABAergic inhibition in the ICX than newly learned responses (Zheng and Knudsen, 1999).

The plasticity of the auditory map in the ICX that is induced by the prism experience is an example of supervised learning, where a visually based instructive signal guides the plasticity (Gutfreund and Knudsen, 2004). The source of the instructive signal to the ICX is the OT (Hyde and Knudsen, 2002). The signal is presumably carried by feedback topographic connections from the OT to the ICX (Hyde and Knudsen, 2000; Luksch et al., 2000). Indeed, the activity of neurons in the ICX can be modulated by visual stimuli (Gutfreund et al., 2002; Bergan and Knudsen, 2009). The visual inputs to the ICX are however strongly gated by GABAergic inhibition in the OT (Gutfreund et al., 2002) and can only be elicited by salient visual stimuli (Bergan and Knudsen, 2009). Visual responses in the ICX are retinotopic and spatially restricted. This makes them ideally suited to provide a template for guiding auditory space representation to match visual space representation through Hebbian learning (Hyde and Knudsen, 2001; Gutfreund et al., 2002; Gutfreund and Knudsen, 2004; Witten et al., 2008). In addition, the highly gated nature of these visual inputs in the ICX is presumably necessary to prevent maladaptive overflow of visual information in the ICX.

The experience-dependent-plasticity in the ICX, as in many other systems, dominates the sensitive period at an early age (Knudsen and Knudsen, 1986; Knudsen, 1998). Prisms that were mounted in owls older than 200 days of age induced very little shifts in the ITD tuning curves of ICX neurons (Knudsen, 1998). More recent experiments, however, have shown that prism experience can modify the auditory space map in the ICX in adult owls. Linkenhoker and Knudsen (2002) demonstrated that incremental training, i.e., using small optical displacements and gradually stepping out, is a powerful strategy to elicit plasticity in adult ICX. A second strategy was to provide a richer sensory environment by allowing the prisms mounted owls to hunt live mice (Bergan et al., 2005). Plasticity was five times greater in the hunting owls compared to the control group that was fed with dead mice. Therefore, plasticity can be induced during adulthood as well, but to a smaller extent and it requires a richer and more rigorous sensory experience than in young owls. Analysis of the anatomical changes in the IC that accompany adult plasticity showed that the changes are similar to those accompanying plasticity in young owls suggesting that in the IC the same basic mechanisms of plasticity take place in adults as well as young owls (Linkenhoker et al., 2005).

## Correlates of response adaptation in the barn owl's inferior colliculus

**Figure 9:**
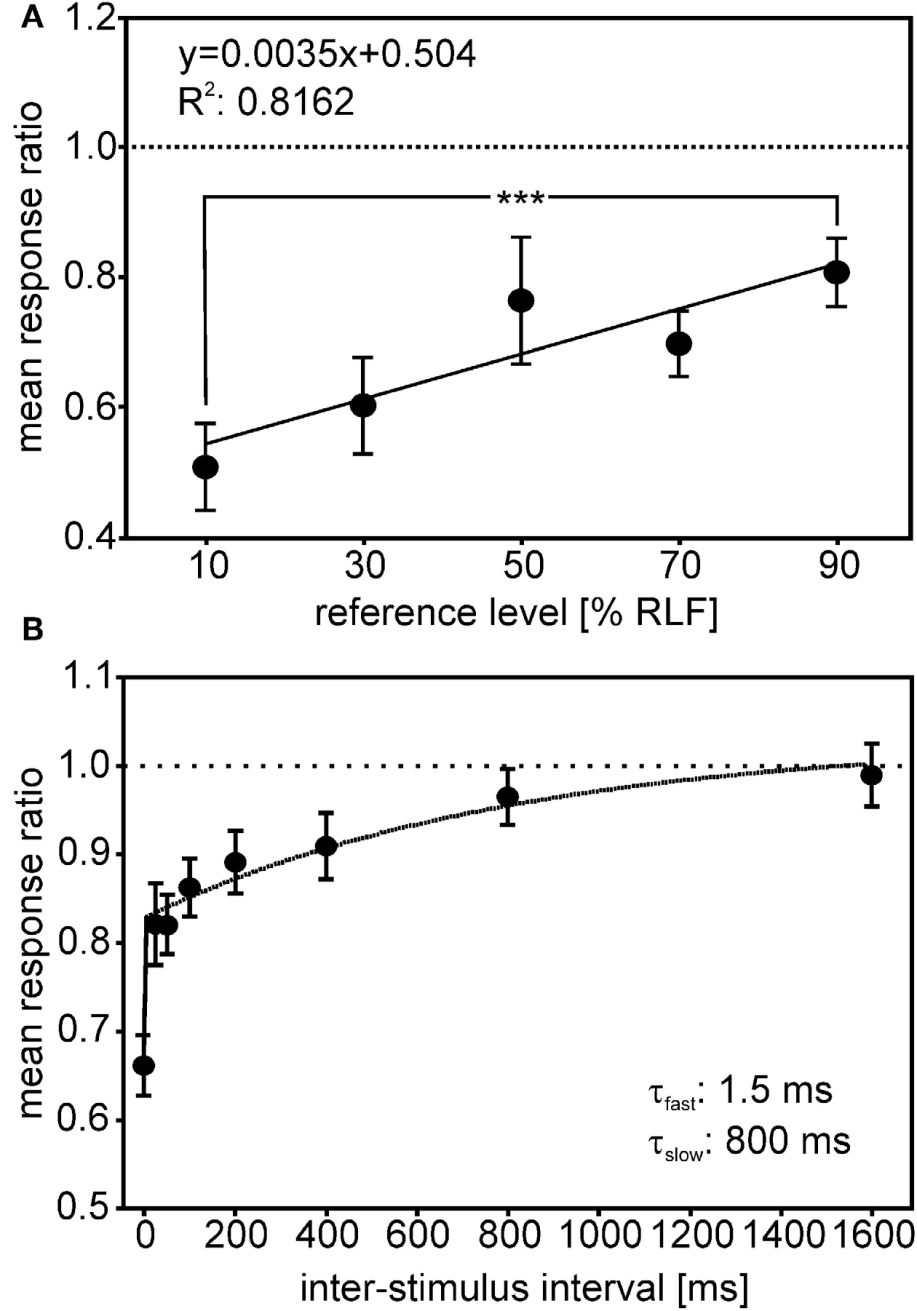
**Adaptation in ICC.** The response ratio, defined as the quotient of the unit's response rate to a given probe (second stimulus), divided by the response rate of a particular reference-stimulus (first stimulus), is plotted. **(A)** The mean response ratios as a function of the relative reference-stimulus level. Probe and reference stimulus had the same level. The reference level refers to the dynamic range as determined from the rate-level function (RLF). The responses ratios decreased with decreasing reference level and differed significantly between 90 and 10% reference-stimulus level (Mann–Whitney test, *P* < 0.0001). **(B)** Responses ratios as a function of the interstimulus interval (ISI) tested. Response ratios were significantly reduced compared with unity for ISIs up to 400 ms (one-sample *t*-tests, all *P* < 0.05; significance levels are indicated as follows: *P* < 0.05: ^*^; *P* < 0.01: ^**^; *P* < 0.001: ^***^). The recovery function could be fitted well (*R*^2^ = 0.982) by a double exponential with a short time constant of 1.25 ms and a long time constant of 800 ms (see inset in **B**).

Two studies have investigated response adaptation in IC neurons. Gutfreund and Knudsen (2006) investigated same- and across frequency adaptation using a double stimulus paradigm in IC neurons. Whereas same-frequency stimulation resulted in relatively strong adaptation of the second stimulus (probe) in reference to the first stimulus (masker), across-frequency adaptation could not be observed in ICCls. Singheiser et al. (2012) investigated response adaptation in ICC with a slightly different double-stimulus paradigm (Figure 9). Singheiser et al. (2012) found weaker response adaptation than Gutfreund and Knudsen (2006) that might be due to monaural stimulation versus binaural stimulation, respectively. The former authors determined the increase in the stimulus level of the probe that was necessary to overcome response adaptation. They found a value of <7 dB that depended on the overall stimulus level of the first stimulus (Figure 9A). Furthermore, by varying the interstimulus interval between first and second stimulus, recovery from adaptation could well be described by a double exponential fit with a fast (1.5 ms) and a slow component of 800 ms (Figure 9B). Wagner (1991) also found very short time constants (about 2 ms) when barn owls were tested to detect brief appearances of ITDs. Recovery functions of Gutfreund and Knudsen (2006) yielded time scales of about 100 ms intermediate between the two components obtained by Singheiser et al. (2012).

Reches and Gutfreund (2008) investigated stimulus-specific adaptation in the auditory pathways of the owl by recording sequences of different stimuli (ITD, ILD, sound level, and frequency) in the ICX, the OT and the arcopallium gaze fields (AGF) in the forebrain. In an stimulus-specific adaptation paradigm, a standard stimulus is presented frequently in comparison to a deviant stimulus. The standard and the deviant stimulus are chosen from tuning curves such that they evoke the same response if they are presented at the same rate. It is tested whether neurons show higher response rates to the deviant than to the standard stimulus. By this context-dependent stimulation neurons involved in novelty-detection can be found. Neurons in ICX showed stimulus-specific adaptation only to frequency but not to the other cues, neurons recorded in OT displayed strong stimulus-specific adaptation to all four types of stimuli. The same was observed for neurons in AGF that were tested with the frequency and ITD cues. Reches and Gutfreund (2008) hypothesized that stimulus-specific adaptation is computed at least twice in the brain, once for frequency, at the level of the IC or below, and once for other auditory features at higher order nuclei. SSA to the frequency of the sound was also reported in the mammalian IC, mostly in broadly tuned units (Malmierca et al., 2009).

## Outlook

The barn owl IC has numerous adaptions linked to neural coding of sound location. In the past four decades many of the underlying morphological and functional specializations have been discovered. In this context, work on the barn owl's IC has often served as role model for studies in other systems. This holds specifically for the map of auditory space, and the role of the IC in plasticity, noise reduction, cross-frequency integration, and multiplicative interactions between orthogonal cues. On the other hand, research on owl IC has profited from both morphological and physiological progress made in studies with other birds and mammals. This include, for example, questions on the functional properties of ICS, the role of envelope, and carrier in responses to ITDs, as well as the function of adaptation in these neurons.

Interestingly, one of the hallmarks of processing in the barn owl's IC, the map of auditory space in ICX, has still not been clearly shown in mammalian IC. Early work by Binns et al. (1992) and Aitkin et al. (1985) suggested that the mammalian IC may contain a map. The significance of these findings remains unclear, however, because no one has followed up these findings. Also, potential homologies between the avian ICX and the mammalian ICX remain unresolved. For example, ICX in mammals contains somatosensory information (Aitkin et al., 1981), which has not been reported for barn owl ICX so far. The clearest evidence for a map in the mammalian IC comes from the work of Schnupp and King (1997), which revealed a map of sound azimuth in the nucleus of the brachium of the IC. Since this nucleus projects topographically to the superior colliculus (King et al., 1998) and in turn receives information from the superior colliculus, the nucleus of the brachium of the IC has many functions analog to the owl ICX. In the pigeon a map of auditory space has been observed in ICX (Lewald, 1988). A map of ITD was found in chicken NL (Köppl and Carr, 2008). These maps are, however, not as sophisticated as the map in the barn owl. For example, the maps in the pigeon and the chicken are maps of azimuth and not of two-dimensional space as in the barn owl. Moreover, in other owl species such as the burrowing owl and the great-horned owl that are not as much adapted to live and hunt in the dark as the barn owl and do not exhibit ear asymmetry the elevational restrictions of receptive fields are less strict than in the barn owl or missing at all (Volman and Konishi, 1990). In this respect, the evolutionary pressure arising from hunting in the dark has shaped IC morphology and physiology in the barn owl.

Another interesting feature of barn owl IC is the existence of a lateral and a medial shell. Many regions have been detected anatomically in chicken IC (Puelles et al., 1994; Wang and Karten, 2010). However, it is not clear which, if any, corresponds to ICCls. To find out more about this, both morphological tracing studies and physiological characterization of these structures will be necessary. The same holds for ICCms. On the other hand, while the barn owl clearly has a superficial subnucleus, the physiological properties of the cells in this structure have not yet been studied.

For barn owl research itself, several challenging questions remain: one is whether the functional arrays that have been identified by subsequent recordings at many positions in dorso-ventral penetrations indeed act in concert. Testing this would require the use of new techniques like multichannel-electrodes recordings. A second question relates to microcircuits: most cells have been quantified morphologically and by extracellular recordings, but intracellular characterizations of IC response properties are largely missing (for exceptions see Peña and Konishi, 2001, 2002) as is the precise synaptic interaction in all of the sub-nuclei. Here, working with slices might bring progress.

This brief review of the current knowledge about the barn owl IC was intended to summarize what is known about this structure, to point out its remarkable adaptation to a specific task, sound localization, and to mention briefly remaining issues. Much more work will be necessary to arrive at a broader and deeper understanding of the barn owl IC as is, for example, available for the mammalian IC.

### Conflict of interest statement

The authors declare that the research was conducted in the absence of any commercial or financial relationships that could be construed as a potential conflict of interest.
